# Na^+^/H^+^ exchanger 1 participates in tobacco disease defence against *Phytophthora parasitica* var. *nicotianae* by affecting vacuolar pH and priming the antioxidative system

**DOI:** 10.1093/jxb/eru351

**Published:** 2014-08-28

**Authors:** Xianyang Chen, Hexigeduleng Bao, Jie Guo, Weitao Jia, Fang Tai, Lingling Nie, Ping Jiang, Juanjuan Feng, Sulian Lv, Yinxin Li

**Affiliations:** Key Laboratory of Plant Molecular Physiology, Institute of Botany, Chinese Academy of Sciences, Beijing 100093, PR China

**Keywords:** Cellular redox homeostasis, disease resistance, NAD(P) (H) pool, *NHX1*, tobacco, vacuolar H^+^ flux and pH.

## Abstract

Despite the importance of NHX1 (Na^+^/H^+^ exchanger 1) in plant salt tolerance, little is known about its other functions. In this study, intriguingly, it was found that NHX1 participated in plant disease defence against *Phytophthora parasitica* var. *nicotianae* (*Ppn*) in *Nicotiana benthamiana*. *NbNHX1* was originally isolated from *N. benthamiana*, and characterized. The subcellular localization of NbNHX1 with its C-terminus fused with green fluorescent protein indicated that NbNHX1 localized primarily to the tonoplast. *Tobacco rattle virus*-induced *NbNHX1* silencing led to reduced H^+^ efflux from the vacuole to cytoplasts, and decreased *Ppn* resistance in *N. benthamiana*. After attack by *Ppn*, *NbNHX1-*silenced plants exhibited impaired ability to scavenge reactive oxidative species (ROS) induced by the pathogen. *Pea early browning virus*-mediated ectopic expression of *SeNHX1* (from *Salicornia europaea*) or *AtNHX1* (from *Arabidopsis thaliana*) both conferred enhanced *Ppn* resistance to *N. benthamiana*, with a lower H_2_O_2_ concentration after *Ppn* inoculation. Further investigation of the role of NHX1 demonstrated that transient overexpression of *NbNHX1* improved the vacuolar pH and cellular ROS level in *N. benthamiana*, which was coupled with an enlarged NAD(P) (H) pool and higher expression of ROS-responsive genes. In contrast, *NbNHX1* silencing led to a lower pH in the vacuole and a lower cellular ROS level in *N. benthamiana*, which was coupled with a decreased NAD(P) (H) pool and decreased expression of ROS-responsive genes. These results suggest that NHX1 is involved in plant disease defence; and regulation of vacuolar pH by NHX1, affecting the cellular oxidation state, primes the antioxidative system which is associated with *Ppn* resistance in tobacco.

## Summary

NbNHX1 affects the cellular pH and oxidation state by regulating the vacuolar H^+^ flux, which primes the antioxidative system associated with *Phytophthora parasitica* var*. nicotianae* resistance in tobacco.

## Introduction

Due to their sessile nature, plants have developed various biochemical and physiological processes to respond to environmental stresses. They efficiently regulate redox homeostasis in response to abiotic and biotic stresses, and the redox state is regarded as one of the most important indicators for evaluating the situation of the cell (Foyer *et al.*, 2009). The components in the plant cell that affect the redox potential and intracellular redox state include O_2_/H_2_O, OH·/H_2_O, oxidized/reduced glutathione (GSSG/GSH), oxidized/reduced NADs [NAD(P)/NAD(P)H], and oxidized/reduced ferredoxin (Fdox/Fdred) (Foyer and Noctor, 2003). Various compartments in plant cells retain redox homeostasis, and a new homeostasis is rapidly established when the original redox state is disrupted by stresses (Sharma and Dietz, 2009). Increasing the redox pool can boost resistance to abiotic or biotic stresses (Chen *et al.*, 2003; Hayashi *et al.*, 2005). The genetic engineering of redox components has been shown to improve disease resistance in plants. For example, overexpression of a glutathione reductase gene in wheat improves the resistance to powdery mildew and induces transcript accumulation of other pathogenesis-related genes (Y.P. Chen *et al.*, 2007). Similarly, overexpression of a gene related to the NAD(P)H pool in rice improves the resistance to hydrogen peroxide (H_2_O_2_) and disease (Hayashi *et al.*, 2005).

The NADPH oxidase (NOX) in the plasma membrane accepts electrons from NADPH at the cytosolic side of the membrane and donates them to molecular oxygen at the other side of the membrane, thus producing superoxide either outside the plasma membrane or in the endosomes (Sato *et al.*, 2001). A major endocytotic route in plants is vesicle trafficking from the plasma membrane to the vacuole that plays an important role in many stresses (Leshem *et al.*, 2006). The membrane trafficking provides the opportunity of endosomes to generate reactive oxygen species (ROS) by a mechanism similar to that in the plasmalemma–apoplast system, based on the activity of NOX (Andreev, 2012). Recently, many pieces of evidence point to the possibility that the tonoplast can generate ROS. Cytochemical visualization displays O_2_^−^ generation in the tonoplast (Romero-Puertas *et al.*, 2004). The proteomics of the tonoplast demonstrate that it contains NOX-like proteins (Carter *et al.*, 2004). Several enzymes associated with ROS generation in the vacuole have also been identified by biochemical analysis (Pradedova *et al.*, 2011).

The vacuole, as the largest endosome, has been confirmed to participate actively in cellular oxidative events. Loss of vacuole function causes sensitivity to oxidative stress in the ﬁssion yeast *Schizosaccharomyces pombe* (Mikawa *et al.*, 2010). ROS can diffuse out of the chloroplast at considerable rates and be transported to the vacuole by intrinsic proteins in the tonoplast (Brunetti *et al.*, 2011). Maturation of endomembrane organelles involves luminal acidification driven by vacuolar H-ATPase and cation/H^+^ exchangers, which are also involved in charge balance during the NOX respiratory bust (El Chemaly *et al.*, 2010). Transmembrane proton transfer in the vacuole is associated with superoxide production inside the endosome, which is dependent on the pH of the compartment (Lamb *et al.*, 2009). Protons transported into the vacuole lumen are consumed in the dismutation of superoxide, which occurs rapidly under acidic conditions, but slows down remarkably under alkaline conditions (Plonka *et al.*, 1986).

Na^+^/H^+^ exchanger 1 (NHX1) localized in the tonoplast affects transmembrane H^+^ flux in the vacuole by sequestering cytoplasmic Na^+^ in the vacuole and pumping vacuolar H^+^ into the cytoplasm (Blumwald, 2000). The function of NHX1 in the plant response to salt stress has been extensively studied. Overexpression of *NHX1* improves the salt tolerance of many plant species, including *Arabidopsis* (Apse *et al.*, 1999), tomato (Zhang and Blumwald, 2001), tall fescue (Tian *et al.*, 2006), maize (M. Chen *et al.*, 2007), and *Nicotiana tabacum* (Zhou *et al.*, 2008). In contrast, the T-DNA insertional mutant of *AtNHX1* leads to stronger sensitivity to NaCl in *Arabidopsis* seedlings (Apse *et al.*, 2003). In addition, NHX1 also has important functions in cellular K^+^/Na^+^ homeostasis (Shabala and Cuin, 2007), vacuolar pH regulation (Yamaguchi *et al.*, 2001), cold tolerance (Li *et al.*, 2007), and regulation of plant growth, flower development, and reproduction (Bassil *et al.*, 2011).

However, little is known about the role of NHX1 in biotic stresses such as pathogen attack. Biotrophic pathogens obtain nutrients from the plant and suppress host defence during the infection (O’Connell and Panstruga, 2006). Thus, the defence of plants against biotrophic pathogens relies on oxidative burst and induces cell death (Glazebrook, 2005). In contrast, necrotrophic pathogens are not restricted by cell death, but rather feed on the remains of dead organisms or their by-products (Glazebrook, 2005). *Phytophthora parasitica* is considered a hemibiotroph, which initially establishes itself in host tissues as a biotroph. It then switches to a necrotrophic type of growth, and rapidly invades and kills the host cells after disease burst which induces production of intracellular ROS in the host cells (Woltz, 1978; Galiana *et al.*, 2005). *Phytophthora parasitica* var. *nicotianae* (*Ppn*) is regarded as one of the most destructive and widespread pathogens that causes black shank disease in tobacco. Thus, investigation of whether NHX1 is associated with plant defence against *Ppn* and responds to oxidative damage caused by *Ppn* in tobacco has gained considerable interest.

In the present work, *NbNHX1* was originally isolated from *Nicotiana benthamiana*. *NbNHX1* silencing led to stronger *Ppn* sensitivity in *N. benthamiana*. The general function of NHX1 on plant defence was confirmed by transformation of *NHX1* from *Salicornia europaea* or *Arabidopsis* improving the *Ppn* resistance in tobacco. Further investigation demonstrated that NHX1 had functions in regulating the pH in the vacuole and cellular ROS level, which could prime the antioxidative system.

## Materials and methods

### Plant material

The seeds of *N. benthamiana* were spread on MS medium (Murashige and Skoog, 1962). After 2 weeks, tobacco seedlings were transferred into plastic pots containing a mixture of vermiculite, turf, and humus (1:1:1; v/v/v), and grown in a greenhouse under the following conditions: 16h light/8h dark photoperiod, 25±2 °C, and 50±10% relative humidity. The plants were watered weekly with half-strength Hoagland nutrient solution (Zhou *et al.*, 2008).

### Isolation of *NbNHX1*

First-strand cDNA was synthesized using total RNA isolated from *N. benthamiana* seedlings by the reverse transcription system (Promega, Madison, WI, USA) according to the manufacturer’s protocol. Full-length cDNA was obtained by using 3' RACE (rapid amplification of cDNA ends) and 5' RACE kits according to the manufacturer’s instructions (Invitrogen, Karlsuhe, Germany). The amplicon was cloned into the cloning vector pEASY-T-Simple (TransGen, China) and sequenced.

The nucleotide sequence, deduced amino acid sequence, and open reading frame encoded by *NbNHX1*, *AtNHX1*, and *SeNHX1* were analysed by DNAman software. Multiple sequence alignment and the rooted phylogenetic tree were performed with ClustalW (http://www.genome.jp/tools/clustalw/). The transmembrane topology prediction was performed using TopPred2 (bioweb.pasteur.fr/seqanal/interfaces/toppred.html).

### Green fluorescent protein (GFP) plasmid construction and microscopy analysis

The coding sequence of *NbNHX1* was amplified and inserted into *Kpn*I/*Bam*HI sites of the pCAMBIA1300-35S::GFP vector to produce pCAMBIA1300-35S::NbNHX1-GFP. Three days after agroinfiltration into leaves of tobaccos, the protoplasts isolated from inoculated leaves were observed on a Zeiss LSM 510 META confocal microscope.

### Generating *NbNHX1*-silenced tobacco

The available sequences of *NHX* genes in *N. benthamiana* were obtained from the website of the ‘Sol genomics network’ (http://solgenomics.net/). It was found that unigene information of *NHX2* (ID: SGN-U515339) and *NHX3* (IDs: SGN-U518331 and SGN-U521516) have been annotated in *N. benthamiana*. However, the unigenes SGN-U518331 and SGN-U521516 are different fragments of the *NHX3* gene, which was confirmed by the expressed sequence tag (EST) sequence of *NHX3* cloned in the present study (Supplementary Fig. S1A available at JXB online). Therefore, a 263bp sequence from *NbNHX1* (nucleotides 1321–1583) was selected as a distinctive sequence after alignment with *NHX2* and *NHX3*, respectively (Supplementary Fig. S1B, C).

*Tobacco rattle virus* (TRV)-induced gene silencinge was used in *N. benthamiana* (Liu *et al.*, 2004). The distinctive sequence from *NbNHX1* described above was constructed into the pTRV2 vector as pTRV2-NbNHX1 (Supplementary Fig. S2A at *JXB* online). Agroinfiltration of 4-week-old *N. benthamiana* plants with pTRV1 was in combination with pTRV2-NbNHX1, pTRV2-PDS, and pTRV2 empty vector. To test whether the TRV clones could induce gene silencing in tobacco plants, the ability of the TRV-VIGS (virus-induced gene silencing) vector to suppress the expression of the endogenous phytoene desaturase gene (*PDS*), which was used as the reporter in the system (Liu *et al.*, 2002), was examined. Four weeks after agroinfiltration, when leaves infected with pTRV2-PDS exhibited bleaching (Supplementary Fig. S2B), the expression of *NbNHX1* was tested in agroinfiltrated tobacco plants by real-time PCR (Supplementary Fig. S4C). The *NbNHX1* primers were: forward primer 5' GTTCAAGAGTTACTACAAGGCACG 3' and reverse primer 5' CAATGGTAATGGTGCTGGTAATC 3'. MxPro software was used to quantify gene expression. TRV-Nb plants as *NbNHX1*-silenced tobacco were created by transformation of pTRV2-NbNHX1; TRV plants as control were created by transformation of pTRV2 empty vector into *N. benthamiana* (Supplementary Fig. S2A, C). Relative expression of *NbNHX1* in TRV-Nb plants was normalized against that in TRV plants. TRV-Nb and TRV plants did not display any difference in growth phenotype after 4 weeks of agroinfiltration.

### Preparation of protoplasts and isolation of vacuoles

The isolation of protoplasts from *N. benthamiana* was based on a previous report (Chen *et al.*, 2011*b*). To release the vacuoles, the solution containing the protoplasts was diluted to a final concentration of 0.2M mannitol with 25mM TRIS-HCl (pH 7.5) and gently pipetted three or four times at 2min intervals for 10min. The suspension was then loaded onto the top of 8% (w/v) Ficoll 400 in 25mM TRIS-HCl (pH 7.5) and 0.5M mannitol. The gradient was centrifuged at 2000 *g* for 30min at 4 °C in a swinging bucket rotor. Vacuoles collected in the top layer were removed and examined using an A/O Spencer Bright Line Hemacytometer with a Nikon inverted phase contrast microscope.

### Measurement of net H^+^ flux with non-invasive micro-test electrophysiological technology (NMT)

Non-invasive measurement of net H^+^ flux in vacuoles using NMT (NMT system, BIO-001A, Younger USA Sci. & Tech. Corp., Amherst, MA, USA) was performed based on a previous report (Chen *et al.*, 2011*b*).

The vacuolar net H^+^ flux was detected in the measuring solution that simulates the intracellular ionic environment (Chen *et al.*, 2011*b*). Prior to the measurement, the solution containing vacuoles was placed at the centre of the coverslip treated with 0.008% (w/v) poly-l-lysine. After vacuoles settled onto the coverslip (~15min), the residual solution was removed with a pipette, and then 3ml of measuring solution [0.05mM MES, 0.5M mannitol, 0.1mM NaCl, 0.1mM CaCl_2_, and 100mM K^+^ (potassium gluconate, C_6_H_11_O_7_K) pH 6.8] was added slowly to the container. The H^+^ flux of the sample vacuole was recorded from 0.5min to 5min under normal conditions. Then, NaCl (1M), KCl (1M), ATP (150mM), or PPi (150mM) stock solution was added slowly to reach the final concentration in the buffer. After the ions stabilized in solution for 1–2min, the H^+^ flux measurement was restarted and continued for a further minute. The mean net H^+^ flux was calculated based on all the transient H^+^ flux data recorded during the period of treatment. The value obtained from NMT indicates net ion flux, and the positive values of ion flux in the figures represent cation efflux from the vacuole into the cytoplast, and vice versa.

### Pathogen challenge

The *Ppn* (race 0, pathogen of black shank disease) was cultured on PDA medium (potato, 200g l^–1^; sucrose, 20g l^–1^; agar, 15g l^–1^; pH 6.5). *Ppn* inoculation was based on the method of Guo *et al.* (2004). When the fungal mycelia had spread throughout the PDA plate, a plug of medium containing the fungal mycelia was removed using a plastic borer. Detached leaves (the third from top of the plant) were used for pathogen challenge. The leaf was wounded with a toothpick; wounds were located on the each side of the main vein. The mycelia were inoculated onto the right side of the main vein at the wound site; the left side of the leaf was used as a wound-only control site (Guo *et al.*, 2004). The wounded leaves were kept in Petri dishes on water-soaked filter paper at 28±2 °C, 16h light/8h dark photoperiod until measurement.

The area of the wilt spot was measured using Matlab software. The range of grey values in the control part of each leaf was first scanned, and then the area with a continuous grey value higher than the maximum value of the control was calculated as the wilt spot area.

The symptoms caused by *Ppn* infection were classified into three ranks based on the areas of the wilt spots (Guo *et al.*, 2004): rank 1, no symptom or area of wilt spots <8cm^2^; rank 2, the area of wilt spots is >8cm^2^ and <12cm^2^; and rank 3, the area of wilt spots is >12cm^2^.

### Oxidative resistance analysis

The leaf discs from *NbNHX1-*silenced or *Se/AtNHX1-YFP* ectopically expressed *N. benthamiana* were detached using a plastic borer. The leaf discs were immersed in half-strength Hoagland nutrient solution with 0, 1, or 10mM methyl viologen (MV) for 2 d. The contents of H_2_O_2_ or total chlorophyll were measured by the method of Chen *et al.* (2011*a*).

### Determination of H_2_O_2_

Leaf tissues from around the infected spots were used for determination of H_2_O_2_ content. The measurement of H_2_O_2_ was based on the peroxide-mediated oxidation of Fe^2+^, followed by the reaction of Fe^3+^ with xylenol orange, according to the method of Bellincampi *et al.* (2000).

### PEBV-mediated ectopic gene expression

Based on a previous study, the *Pea early browning virus* (PEBV) system was used to mediate ectopic expression of *SeNHX1* (identified from *S. europaea*, GenBank accession no. AY131235.1) and *AtNHX1* (identified from *Arabidopsis*, TAIR accession no. AT5G27150.1) in *N. benthamiana* (Chen *et al.*, 2011*a*). Two pCAPE2 derivative clones (pCAPE2-SeNHX1 or AtNHX1) were prepared using pCAPE2-YFP as the cloning vector (Supplementary Fig. S2E at *JXB* online). Agroinfiltration of 4-week-old *N. benthamiana* plants with pCAPE1 was in combination with pCAPE2-SeNHX1-YFP, pCAPE2-AtNHX1-YFP, pCAPE2-YFP, and pCAPE2-PDS, while pCAPE2-PDS was used as the positive control (Constantin *et al.*, 2004). Four weeks after agroinfiltration, when leaves of the plants infected with pCAPE2-PDS appeared bleached (Supplementary Fig. S4F), pCAPE2-SeNHX1-YFP (Se-YFP)- and pCAPE2-AtNHX1-YFP (At-YFP)-transformed tobacco plants were ready for further study. PEBV-mediated SeNHX1–YFP, AtNHX1–YFP, and YFP expression in *N. benthamiana* was first observed using a fluorescence microscope, and the expression of *NHX1* genes in *N. benthamiana* was confirmed by real-time PCR (Supplementary Fig. S2G). The primers for *NbNHX1* and *SeNHX1* were: forward primer 5' CAGGTAAAAAAGAAGCAATTCTTCC 3' and reverse primer 5' GAATCGCATTGAAAAGCACCACCGA 3'. The primers for *NbNHX1* and *AtNHX1* were: forward primer 5' CATCTTCTCGTCTTTAGTGAAG 3' and reverse primer 5'CAATGTCCAACTTCTTAAAGAA 3'. Relative expression of *NHX1* in Se/At-YFP plants was normalized against that in YFP plants.

### Measurement of vacuolar pH

Vacuolar pH was measured using the pH-sensitive dye 2',7'-difluorofluorescein (Oregon Green 488) (Wilson *et al.*, 1998). Oregon Green 488 has spectral properties which allow dual excitation at 490nm (the pH-dependent wavelength near its absorption maximum) and at 440nm (the second wavelength, relatively pH independent near to its isobestic point); single emissions between 525nm and 550nm were collected for each excitation wavelength. The fluorescence intensity at an excitation of 488nm against that at 458nm indicates the pH *in situ* (Wilson *et al.*, 1998).

For the calibration curve, before pH staining and detection, the epidermis of leaves was detached and incubated in equilibration buffer (half-strength Hoagland nutrient solution, containing 50mM HEPES or MES, 50mM ammonium acetate, pH 5.0–7.0) for 1h. Dye fluorescence images were collected using a confocal microscope (Zeiss, LSM 510 META) after excitation at 458nm and 488nm, and analysed by Image J (National Institutes of Health). The calibration curve indicating the 488/458 ratio was correlated with the pH in the cell (Supplementary Fig. S3A at *JXB* online).

For measurement of the vacuolar pH in *N. benthamiana*, the epidermis of leaves was detached and incubated in half-strength Hoagland nutrient solution, containing 50mM MES (pH 6.0), 20 μM Oregon Green 488 (Molecular Probes), for 1h in darkness at 23 °C. The pH value was calculated based on the488/458 ratio, using the calibration curve. For the ratio image, colour applied to the drawing was generated by Matlab, which converted the ratio of the grey value in each picture pixel at an excitation wavelength of 488nm against that at 458nm to a specific colour.

### Flow cytometric analysis

The GFP and CellROX Deep Red (DR) fluorescence in the protoplasts was detected or screened by a MoFlo XDP high-speed flow cytometer (Beckman-Coulter, USA) with a 70 μm ceramic nozzle at 60 psi sheath pressure. The biparametrically analysed outputs were shown as dot plots in which the viable cell populations were gated based on forward and side scatter (FSC) values. GFP fluorescence was excited with 488nm, and detected with a 530nm band-pass filter; and DR fluorescence was excited with 640nm, and detected at 670nm (http://www.lifetechnologies.com/order/catalog/product/C10422#). The positive protoplasts with green or DR fluorescence were screened by selecting box R6 or R7, in which a near zero percentage (<1% in the present study) of control protoplasts (which completed the transfection procedure without plasmids or without staining) showed green or DR fluorescence. The average fluorescence intensity was obtained automatically from flow cytometry.

### Measurement of nicotinamide coenzymes

Leaves (the third leaf from the top of the plant) of tobacco seedlings were detached for measurements. The contents of nicotinamide coenzymes were determined as described by Hayashi *et al.* (2005). Tobacco leaves homogenized with 0.1M HCl (for NAD and NADP) or 0.1M NaOH (for NADH and NADPH) at 95 °C were cooled, and then the pH was adjusted to 6.5 for NAD and NADP, or to 7.5 for NADH and NADPH. For NAD and NADH measurements, samples were added to the reaction mixture containing 50mM glycylglycine (pH 7.4), 20mM nicotinamide, 1mM phenazine methosulphate, 1mM thiazolyl blue, and 40 μg ml^–1^ alcohol dehydrogenase. For NADP and NADPH measurements, the samples were added to a reaction mixture containing 50mM glycylglycine (pH 7.4), 20mM nicotinamide, 1mM phenazine methosulphate, 1mM thiazolyl blue, and 2mM glucose-6-phosphate. The reaction mixture was measured in a UV-visible spectrophotometer at 570nm.

### The expression of ROS-responsive genes

ROS-responsive genes were selected from the unigene database in the Sol genomics network (http://solgenomics.net/). The expression patterns of the selected genes in each tobacco plant were analysed by real-time PCR based on the primers described in Supplementary Table S1 at *JXB* online.

### Statistical analysis

One-way analysis of variance (ANOVA) in the SPSS 13.0 statistical package was used for statistical analysis. SE indicatess the standard error, and the repetitions are indicated in every experiment. The significance was tested using the least significant difference (LSD) at the 5% level. Asterisks indicate significant differences from the control in the same treatment at *P*≤0.05.

## Results

### NbNHX1 as an Na^+^/H^+^ exchanger localized in the tonoplast regulated vacuolar H^+^ flux in tobacco

**Fig. 1.**
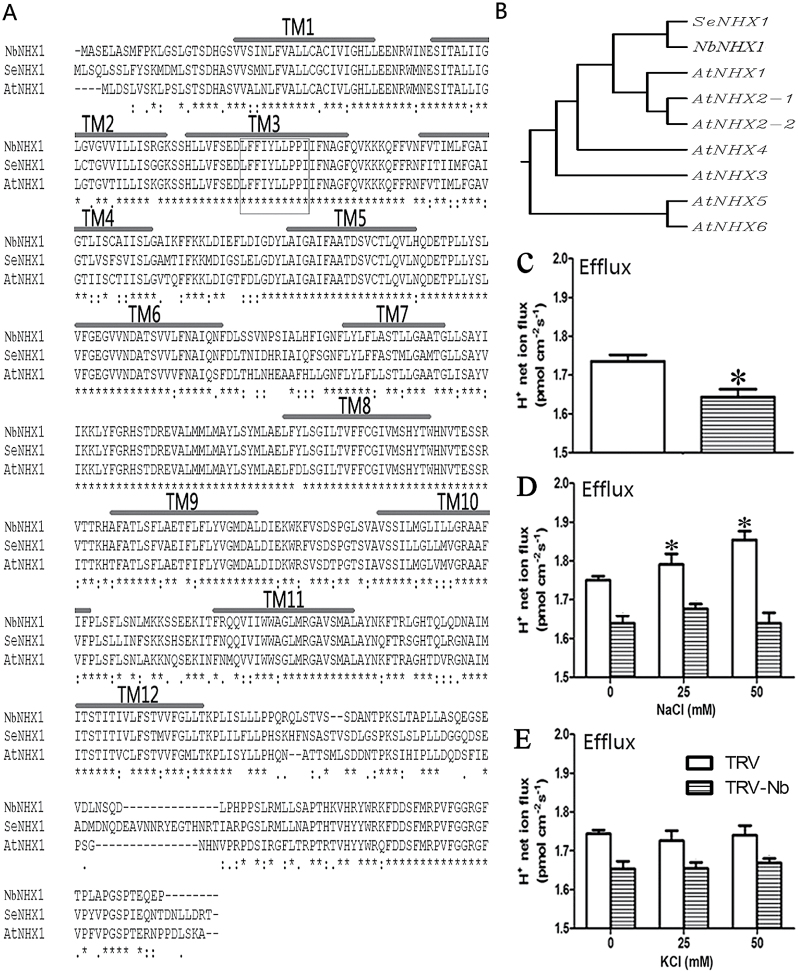
Characterizations of NbNHX1. (A) The alignment of the deduced amino acid sequences of NbNHX1, SeNHX1, and AtNHX1. The box indicates a putative amiloride binding site. NHX1 proteins contain 12 transmembrane domains which are indicated as TM1–TM12. (B) Phylogenetic analysis of *NbNHX1*, *SeNHX1*, and the *NHX* family in *Arabidopsis*. The *NHX* family (with their TAIR accession numbers) is as follows: *AtNHX2-1* (AT3G05030.1); *AtNHX2-2* (AT3G05030.2); *AtNHX3* (AT5G55470); *AtNHX4* (AT3G06370); *AtNHX5* (AT1G54370); *AtNHX6* (AT1G79610). (C) Vacuolar H^+^ net flux in *NbNHX1*-silenced tobacco plants. Mean H^+^ net fluxes for a period of 12min. (D, E) Mean H^+^ net fluxes in vacuoles supplied with 0, 25, and 50mM NaCl (D) or KCl (E). The value obtained from NMT indicates net ion flux, and the positive values of ion flux in the figures represent cation efflux from the vacuole into the cytoplast, and vice versa. Data are means ±SE (*n* = 36 vacuoles from six independent *NbNHX1*-silenced lines). The asterisks on the bars indicate significant differences from the TRV plants (C) or untreated plants (D and E) in the same treatment at *P*≤0.05.

*NbNHX1* (GenBank accession no. JX987081) was originally cloned from *N. benthamiana*, and encodes a polypeptide of 536 amino acids. Hydropathy plot analysis showed that NbNHX1 is similar to SeNHX1 (from *S. europaea*) as well as AtNHX1 (from *Arabidopsis thaliana*) containing 12 transmembrane domains typical for a vacuolar-type Na^+^/H^+^ antiporter. All three amino acid sequences contain a conserved putative binding site for amiloride (‘LFFIYLLPPI’) (Fig. 1A), which is an inhibitor of NHX1 activity (Qiu *et al.*, 2004). The phylogenetic analysis showed that *NbNHX1* and *SeNHX1* are more closely related to *AtNHX1* compared with other *NHX* members in *Arabidopsis* (Fig. 1B).

**Fig. 2.**
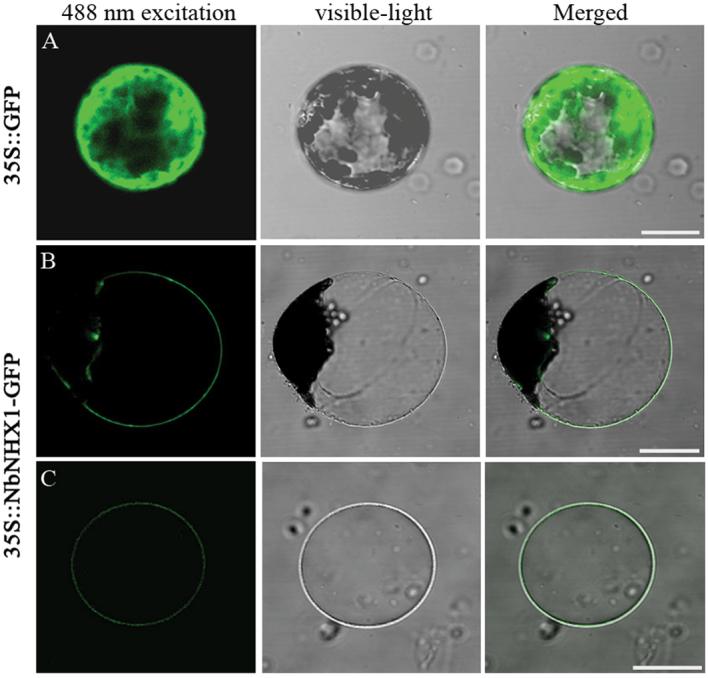
Subcellular localization of NbNHX1 (scale bar=20 μm). (A) Expression of 35S::GFP in protoplasts isolated from leaves of *N. benthamiana*. (B, C) Expression of 35S::NbNHX1-GFP in protoplasts (B) and vacuole (C) from leaves of *N. benthamiana*. (This figure is available in colour at *JXB* online.)

To detect the subcellular localization of NbNHX1, the fusion protein of NbNHX1 with GFP at the C-terminus was expressed in protoplasts of *N. benthamiana*. NbNHX1–GFP exhibited fluorescence in the endomembrane of the vacuolar membranous invagination, with discontinuous fluorescence in the cellular contour (Fig. 2B). Isolated vacuoles from 35S::NbNHX1-GFP-transformed tobacco showed obvious fluorescence in the tonoplast (Fig. 2C). These results indicated that NbNHX1 localized primarily in the tonoplast.

*NbNHX1*-silenced *N. benthamiana* (TRV-Nb plants) were created by a TRV-VIGS approach; TRV plants as control were created by transformation of the pTRV2 vector into *N. benthamiana* (Supplementary Fig. S2A at *JXB* online). Six *NbNHX1*-silenced *N. benthamiana* plants (TN1–TN6) were used to detect vacuolar H^+^ net flux; the results showed a dose-dependent effect of *NHX1* silencing on the decrease in net vacuolal H^+^ efflux (Supplementary Fig. S4B). The average vacuolar net H^+^ efflux decreased to 1.65 pmol m^–2^ s^–1^ in TRV-Nb plants compared with 1.74 pmol m^–2^ s^–1^ in TRV plants (Fig. 1C). To confirm the association between NbNHX1 as an Na^+^/H^+^ exchanger 1 and the increased H^+^ efflux, NaCl and KCl were added into the measuring buffer with a concentration gradient. The vacuolar H^+^ efflux in TRV plants increased under NaCl treatment, whereas that in TRV-Nb plants remained unchanged with the application of NaCl and KCl (Fig. 1D, E).

It is reported that V-ATPase, PPase, and NHX1 act synergistically in the plant vacuole (Blumwald, 2000). Therefore, it was investigated whether the activity of V-ATPase changed in *NbNHX1*-silenced plants. Based on a previous study, ΔH^+^ flux in the vacuole (the change of vacuolar H^+^ flux) after 1.5mM ATP or PPi supply was used to indicate the activities of V-ATPase and PPase in the tonoplast, respectively (Chen *et al.*, 2011*b*). As shown in Supplementary Fig. S5 at *JXB* online, ΔH^+^ flux in the vacuole was comparable between TRV and TRV-Nb plants after 1.5mM ATP or PPi supply, indicating that *NbNHX1* silencing did not impact the activities of V-ATPase and PPase.

### Silencing of endogenous *NbNHX1* decreased *Ppn* resistance in tobacco

**Fig. 3.**
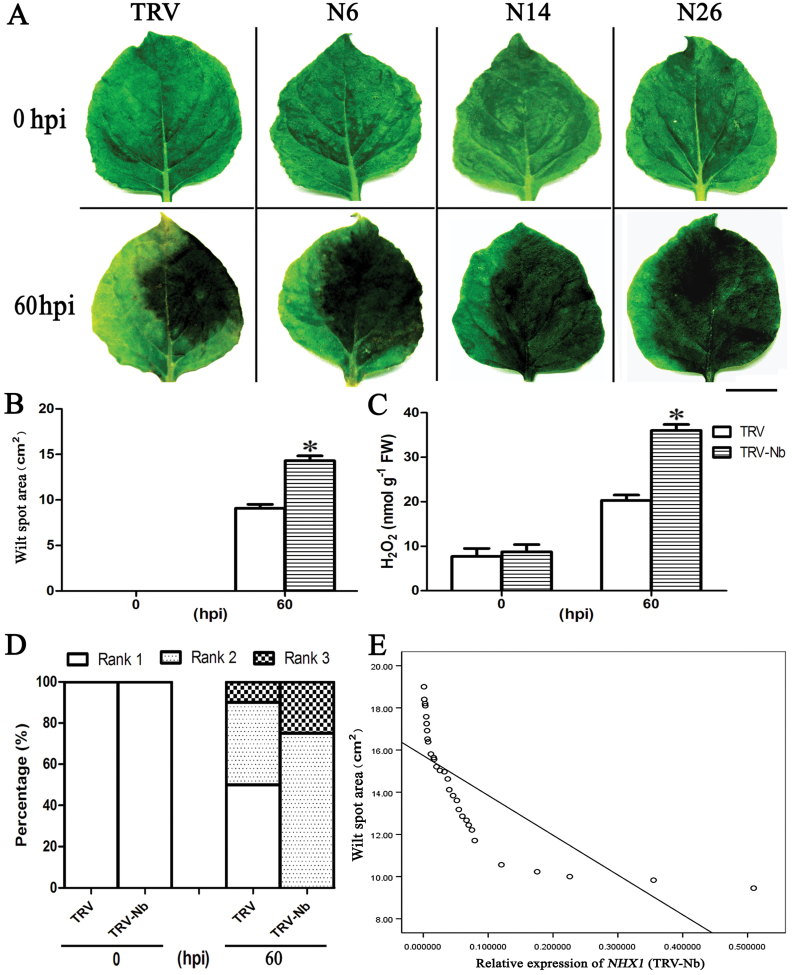
Comparison of disease development between *NbNHX1*-silenced and control tobacco plants. (A) Symptoms of TRV plants (control) and three *NbNHX1*-silenced seedlings at 0 and 60 hpi after *Ppn* inoculation. N6/14/26 represents tobacco seedlings with different expression of *NbNHX1* based on Supplementary Fig. S2C at *JXB* online. Wounding-only treatment on the left side of the leaf served as a control (scale bar=2cm). (B–D) Area of wilt spots (B), H_2_O_2_ content (C), and development of disease course (D) after *Ppn* infection. (E) Correlation analysis between *NHX1* expression and wilt spot area in *NbNHX1*-silenced tobaccos. Data are means ±SE (*n*=30 leaves from 30 independent *NbNHX1*-silenced lines). The asterisks on the bars indicate significant differences from the TRV plants in the same treatment at *P*≤0.05. (This figure is available in colour at *JXB* online.)

Thirty TRV-Nb plants with different expression levels of *NbNHX1* were selected for *Ppn* inoculation (Supplementary Fig. S2C at *JXB* online). No wilt spots were observed in TRV-Nb and TRV plants at 0 hours post-inoculation (hpi), whereas all the leaves exhibited water-soaked wilt after *Ppn* infection (Fig. 3A). At 60 hpi, TRV-Nb plants exhibited more serious disease symptoms and accumulated more H_2_O_2_ than the TRV plants (Fig. 3A–D). Moreover, among the 30 TRV-Nb plants, seedlings with lower *NHX1* expression were more sensitive to *Ppn* (Fig. 3A; Supplementary Fig. S2C, D). The correlation analysis showed that the *Ppn* resistance was positively correlated with *NHX1* expression in TRV-Nb plants, and the correlation coefficient was –0.764 (*P*<0.01) (Fig. 3E).

### Transformation of *SeNHX1* and *AtNHX1* improved *Ppn* resistance in tobacco

*NHX1* genes from *S. europaea* and *Arabidopsis* (*SeNHX1* and *AtNHX1*) were constructed into a viral vector (Supplementary Fig. S2E at *JXB* online), and then expressed in *N. benthamiana* to create ectopic expression of *SeNHX1* or *AtNHX1* tobacco plants (Se-YFP or At-YFP plants) by using the PEBV-mediated ectopic gene expression system. Their controls (YFP plants) were created by transformation of pCAPE2-YFP vector into *N. benthamiana* (Supplementary Fig. S2E). Thirty tobacco seedlings with different expression levels of the *NHX1* gene were selected for further study (Supplementary Fig. S2G).

**Fig. 4.**
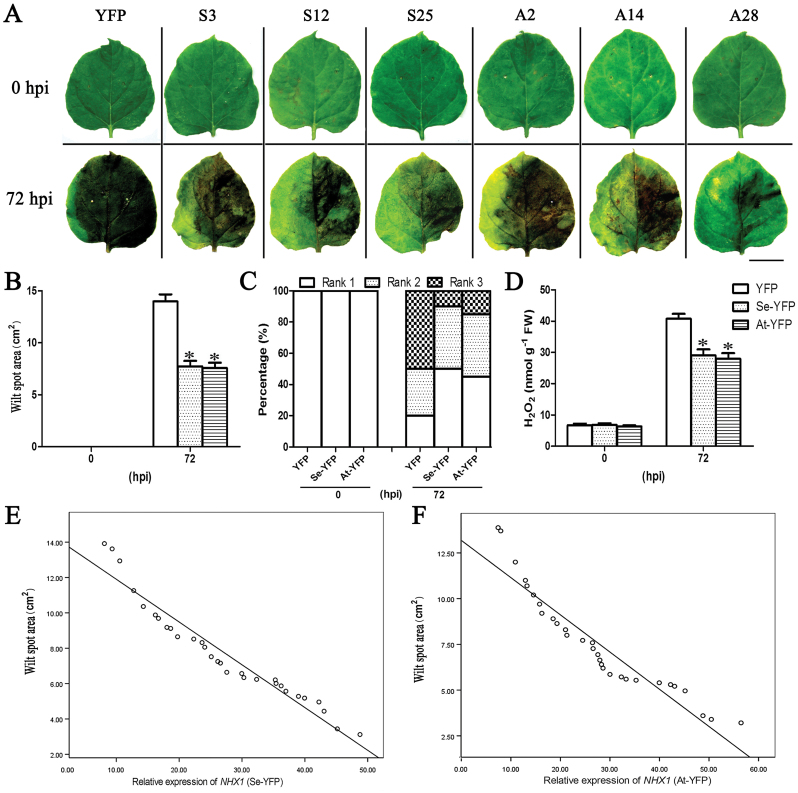
Disease development in *Se/AtNHX1* transgenic plants after *Ppn* inoculation. (A) Disease symptoms on tobacco leaves at 0 and 72 hpi. Wounding-only treatment on the left side of the leaf served as a control (scale bar=2cm). S3/12/25 and A2/14/28 represent tobacco seedlings with different expression of *NHX1* based on Supplementary Fig. S2G at *JXB* online. (B–D) Area of wilt spots (B), development of disease course (C), and H_2_O_2_ content (D). (E, F) Correlation analysis between *NHX1* expression and wilt spot area in *SeNHX1* transgenic tobacco (E) and *AtNHX1* transgenic tobacco (F). YFP indicates pCAPE2-YFP vector-transformed tobacco plants as control and Se/At-YFP indicates pCAPE2-Se/AtNHX1-YFP vector-transformed tobacco plants. Data are means ±SE (*n*=30 leaves from 30 independent *SeNHX1* and *AtNHX1* transgenic lines, respectively). The asterisks on the bars indicate significant differences from the YFP plants in the same treatment at *P*≤0.05. (This figure is available in colour at *JXB* online.)

There were no wilt spots among YFP plants and Se/At-YFP plants before *Ppn* inoculation. At 72 hpi, all tobacco leaves exhibited disease symptoms, while Se/At-YFP plants showed smaller wilt spots and lower H_2_O_2_ contents compared with YFP plants (Fig. 4A–D). Among the 30 transgenic tobacco plants, those with higher *NHX1* expression displayed stronger *Ppn* resistance (Fig. 4A; Supplementary Fig. S4G, H at *JXB* online). The correlation coefficient between the expression of *NHX1* and the wilt spot area was –0.957 (*P*<0.01) in Se-YFP plants and –0.938 (*P*<0.01) in At-YFP plants (Fig. 4E, F).

### NHX1 was associated with oxidative resistance in *N. benthamiana*

**Fig. 5.**
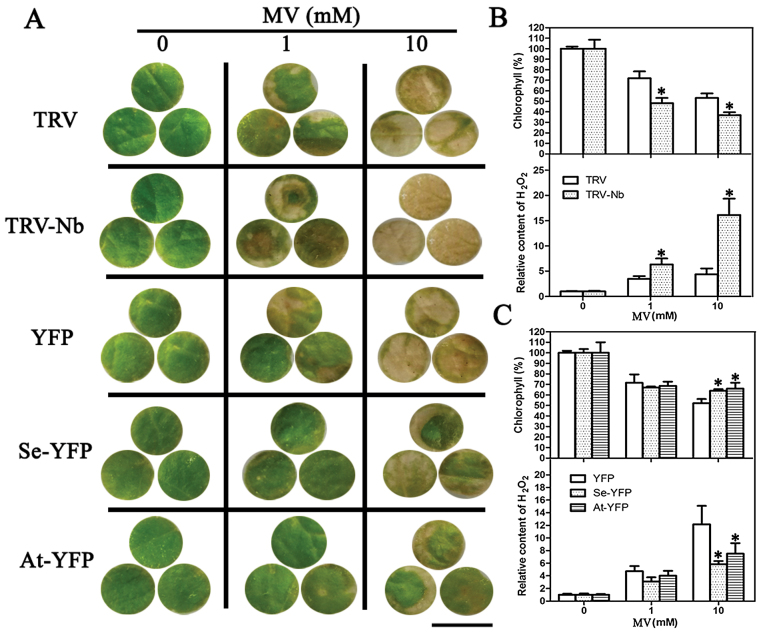
Response of *NbNHX1*-silenced or *At/SeNHX1* ectopically expressed *N. benthamiana* to MV. (A) Leaf discs from *N. benthamiana* were treated with different concentrations of MV (0, 1, and 10mM) for 2 d (scale bars=1cm). The total chlorophyll and H_2_O_2_ contents were detected in *NbNHX1*-silenced plants (B) or *At/SeNHX1* ectopically expressed plants (C), and are expressed as fold changes compared with the value in 0mM MV treatment. TRV represents pTRV2 empty vector-transformed tobacco (control plants), TRV-Nb represents pTRV2-NbNHX1 vector-transformed tobacco (*NbNHX1*-silenced plants), YFP indicates pCAPE2-YFP vector-transformed tobacco as control, and Se/At-YFP indicates pCAPE2-At/SeNHX1-YFP vector-transformed tobacco. Data are means ±SE (*n*=36 leaf discs of 12 leaves from six independent *NbNHX1*-silenced lines or *SeNHX1* and *AtNHX1* transgenic lines, respectively). The asterisks on the bars indicate significant differences from TRV or YFP plants in the same treatment at *P*≤0.05. (This figure is available in colour at *JXB* online.)

Six *NbNHX1*-silenced or *Se/AtNHX1*-YFP ectopically expressed *N. benthamiana* with different expression levels of *NHX1* were selected for analysis of oxidative resistance (Supplementary Fig. S4C–E at *JXB* online). The detached leaf discs were treated with 0, 1, or 10mM MV for 2 d. Without MV treatment, there was no difference in H_2_O_2_ and total chlorophyll contents between TRV and TRV-Nb plants, or among YFP, Se-YFP, and At-YFP plants. However, the TRV-Nb plants exhibited greater H_2_O_2_ and lower total chlorophyll contents than TRV plants under 1mM or 10mM MV treatment (Fig. 5A, B). Although there were comparable H_2_O_2_ and total chlorophyll contents among YFP, Se-YFP, and At-YFP plants under 1mM MV treatment, Se-YFP and At-YFP plants exhibited lower H_2_O_2_ and higher total chlorophyll contents than YFP plants under 10mM MV treatment (Fig. 5A, C).

### NbNHX1 regulated the pH in vacuole

*NbNHX1*-overexpressing plants (Nb-GFP) were created by transformation of the pCAMBIA1300-35S::NbNHX1-GFP vector into *N. benthamiana* as well as their controls (GFP plants) transformed with pCAMBIA1300-35S::GFP. The pH in vacuoles of GFP plants and Nb-GFP plants, or of TRV and TRV-Nb plants was detected by using the ratiometric fluorescein-based pH sensitive dye, 2',7'-difluorofluorescein (Oregon Green 488), in an image-based approach (Wilson *et al.*, 1998). Six *NbNHX1*-overexpressing (NOE1–NOE6) or silenced (NS1–NS6) plants with different expression levels of *NbNHX1* were selected for further analysis (Supplementary Fig. S4C, F at *JXB* online).

First, a calibration curve was created, which was used to quantify the vacuolar pH (Supplementary Fig. S3A at *JXB* online). Then positive control treatments were carried out to validate that the pH quantification method used here worked well. The epidermis of tobacco leaves was detached and incubated in acidic solution (half-strength Hoagland nutrient solution, containing 50mM MES and 50mM ammonium acetate) with pH 5.1 (TEST1) or 5.4 (TEST2) for 1h. Then, the vacuolar pH was calculated based on the calibration curve. As shown in Supplementary Fig. S3B, after staining by Oregon Green 488, the vacuolar pH was 5.12±0.057 in TEST1 and 5.38±0.035 in TEST2, indicating that the quantification method was appropriate for leaves of *N. benthamiana*.

**Fig. 6.**
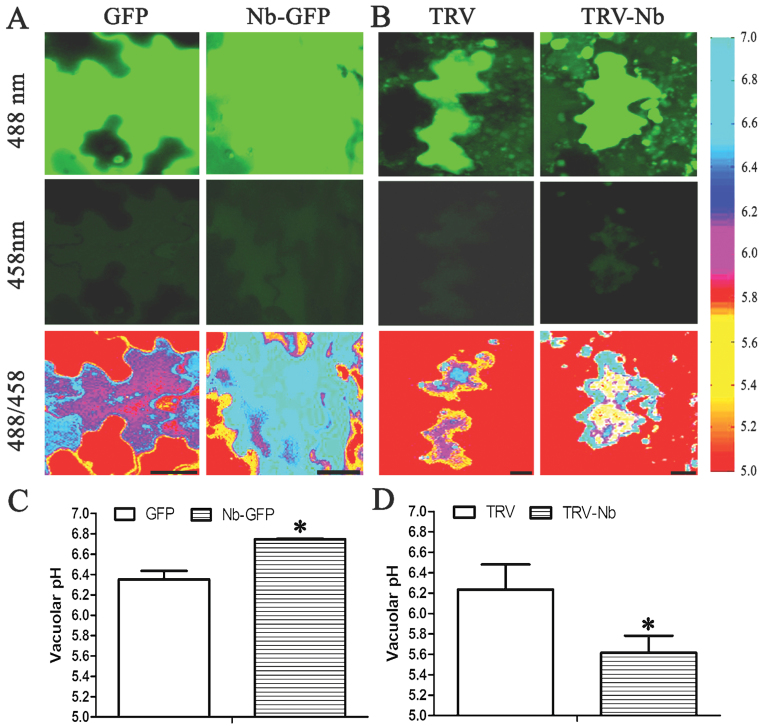
Vacuolar pH in epidermal cells of *N. benthamiana* leaves. (A, B) Ratio images indicating the vacuolar pH in epidermal cells of GFP and Nb-GFP tobacco leaves (A), or TRV and TRV-Nb tobacco leaves (B). Scale bars=20 μm. (C, D) Vacuolar pH quantification in GFP and Nb-GFP tobacco plants (C), or TRV and TRV-Nb tobacco plants (D). GFP indicates pCAMBIA1300-35S::GFP vector-transformed tobacco (control plants), Nb-GFP indicates pCAMBIA1300-35S::NbNHX1-GFP vector-transformed tobacco (*NbNHX1*-overexpressing plants), TRV represents pTRV2 empty vector-transformed tobacco (control plants), and TRV-Nb indicates pTRV2-NbNHX1 vector-transformed tobacco (*NbNHX1* silenced plants). Data are means ±SE (*n* = 360 cells from 36 leaves of six independent *NbNHX1*-silenced or overexpressing lines, respectively). The asterisks on the bars indicate significant differences from the control plants in the same treatment at *P*≤0.05. (This figure is available in colour at *JXB* online.)

The pH of the leaf epidermal cells was shown *in situ* via ratio colour images by using the Matlab software, which showed that the pH of vacuolar zones in Nb-GFP plants was higher than that of GFP plants (Fig. 6A). The calculated vacuolar pH was 6.75 in Nb-GFP plants, which was significantly higher than the pH of 6.36 in GFP plants (Fig. 6C). In contrast, the ratio image showed that the pH of vacuolar zones in TRV-Nb plants was lower than that of TRV plants (Fig. 6B), and the vacuolar pH of 5.61 in TRV-Nb plants was significantly lower than the pH of 6.24 in TRV plants (Fig. 6D).

### NbNHX1 affected the cellular oxidation level

It has been reported that the vacuolar pH is associated with ROS generation (Lamb *et al.*, 2009). Therefore, DR dye was used to monitor the cellular oxidation levels in protoplasts of GFP and Nb-GFP plants, as well as TRV and TRV-Nb plants via flow cytometry, which can precisely detect subtle fluctuation of the cellular oxidation level in high-throughput mode. Six *NbNHX1*-overexpressing plants (Nb-GFP-1, Nb-GFP-3, Nb-GFP-5, Nb-GFP-6, Nb-GFP-8, and Nb-GFP-9, Supplementary Fig. S4G at *JXB* online) and six *NbNHX1*-silenced plants (TRV-Nb-4, TRV-Nb-10, TRV-Nb-12, TRV-Nb-16, TRV-Nb-22, and TRV-Nb-25, Supplementary Fig. S2C) were selected for further study.

**Fig. 7.**
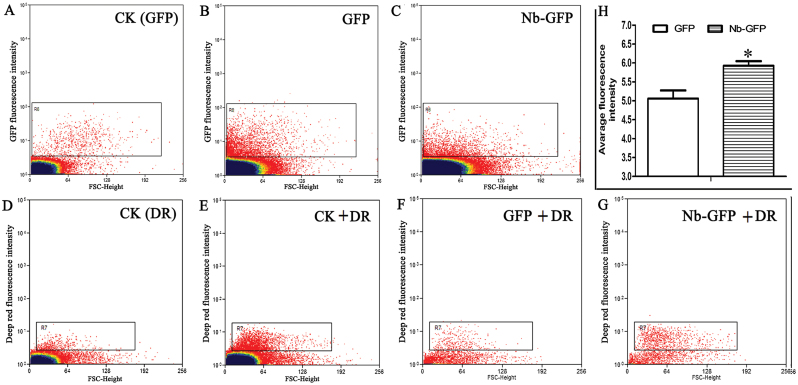
Flow cytometric analysis of the cellular oxidation level in GFP and Nb-GFP tobacco plants. (A–C) Flow cytometric analysis on protoplasts with fluorescence in control plants (A), GFP plants (B), and Nb-GFP plants (C). Biparametric outputs are displayed by the intensity of fluorescence and FSC (forward and side scatter values). The select boxes R6 or R7 are set so that near zero levels (<1%) of control protoplasts show fluorescence. (D, E) Protoplast without DR treatment (D), or with DR treatment (E). (F, G) Protoplasts screened by R6 are analysed on the DR fluorescence level by R7. (H) Average fluorescence intensity of the protoplasts in (F) and (G) screened by R7. Data are means ±SE (*n*=6 batches of protoplasts from six independent *NbNHX1*-overexpressing lines). The asterisks on the bars indicate significant differences from the GFP plants in the same treatment at *P*≤0.05. (This figure is available in colour at *JXB* online.)

For GFP and Nb-GFP plants, the protoplasts expressing green fluorescence should first be screened. According to the MoFlo XDP manual, the select box R6 was set so that <1% of control protoplasts showed fluorescence (Fig. 7A), and then the protoplasts from GFP or Nb-GFP plants in R6 were regarded as positive cells transformed successfully, and were used for further analysis (Fig. 7B, C). Similarly, the select box R7 was set so that <1% of protoplasts showed fluorescence without DR staining (Fig. 7D); the protoplasts after DR staining in R7 were regarded as cells stained successfully (Fig. 7E). Then, the protoplasts from GFP and Nb-GFP plants screened by R6 (Fig. 7B, C) were investigated for DR staining using the R7 select box (Fig. 7F, G). Upon counting the protoplasts in R7, Nb-GFP plants showed increased average fluorescence intensity compared with the GFP plants (Fig. 7H).

**Fig. 8.**
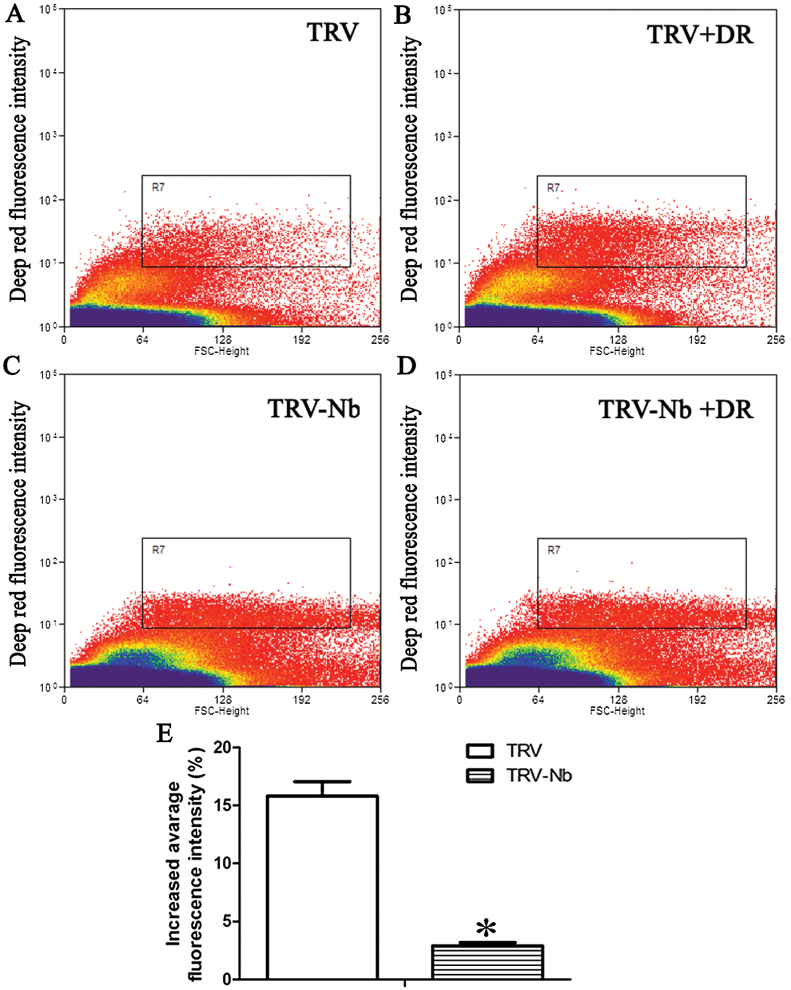
Flow cytometric analysis of the cellular oxidation level in TRV and TRV-Nb tobacco plants. (A–D) Flow cytometric analysis of protoplasts without DR treatment in TRV (A) and TRV-Nb (C); and protoplasts with DR treatment in TRV (B) and TRV-Nb (D). Protoplasts are displayed in biparametric outputs with intensity of fluorescence and FSC. The R7 box is set so that near zero levels (<1%) of control protoplasts show fluorescence. (E) Increased average fluorescence intensity of protoplasts in TRV and TRV-Nb plants. The increased average fluorescence intensity (%) was calculated as follows: [(fluorescence intensity of R7 in protoplasts with DR treatment/fluorescence intensity of R7 in protoplasts without DR treatment)–1]×100%. Data are means ±SE (*n*=6 batches of protoplasts from six independent *NbNHX1*-silenced lines). The asterisks on the bars indicate significant differences from the TRV plants in the same treatment at *P*≤0.05. (This figure is available in colour at *JXB* online.)

Similarly, the cellular oxidation level was also analysed in protoplasts from TRV and TRV-Nb tobaccos by flow cytometry. The average fluorescence intensity increased by 17% in TRV tobacco after DR staining (Fig. 8A, B, E), whereas an increase of only 3% was observed in TRV-Nb plants (Fig. 8C, D, E), which suggested that *NbNHX1* silencing led to a decreased cellular oxidation level in *N. benthamiana*.

### NbNHX1 was involved in cellular redox homeostasis

**Fig. 9.**
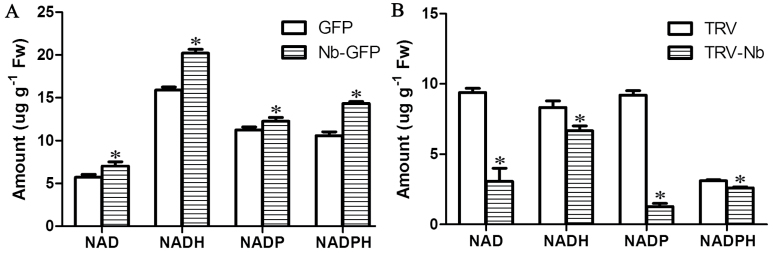
Measurement of the NAD(P) (H) pool. (A) The contents of NAD(P) (H) components in GFP and Nb-GFP plants. (B) The contents of NAD(P) (H) components in TRV and TRV-Nb plants. Data are means ±SE (*n*=6 leaves from six independent *NbNHX1* silenced or overexpressing lines, respectively). The asterisks on the bars indicate significant differences from the control plants in the same treatment at *P*≤0.05.

To investigate whether overexpression or silencing of *NbNHX1* could affect the cellular NADPH homeostasis, the stability of the NAD(P) (H) pool indicating redox homeostasis was calculated in GFP and Nb-GFP (Nb-GFP-1, Nb-GFP-3, Nb-GFP-5, Nb-GFP-6, Nb-GFP-8, and Nb-GFP-9, Supplementary Fig. S4G at *JXB* online) plants, as well as TRV and TRV-Nb (TRV-Nb-4, TRV-Nb-10, TRV-Nb-12, TRV-Nb-16, TRV-Nb-22, and TRV-Nb-25, Supplementary Fig. S2C) plants. Compared with the GFP plants, the Nb-GFP plants exhibited higher contents of NAD(P) (H) components (Fig. 9A), resulting in a significant increase in the NAD(P) (H) pool [NAD(P)+NAD(P)H]. In contrast, silencing of *NbNHX1* decreased the contents of NAD(P) (H) components and the NAD(P) (H) pool in tobacco plants (Fig. 9B).

### NbNHX1 regulated the expression of ROS-responsive genes

**Fig. 10.**
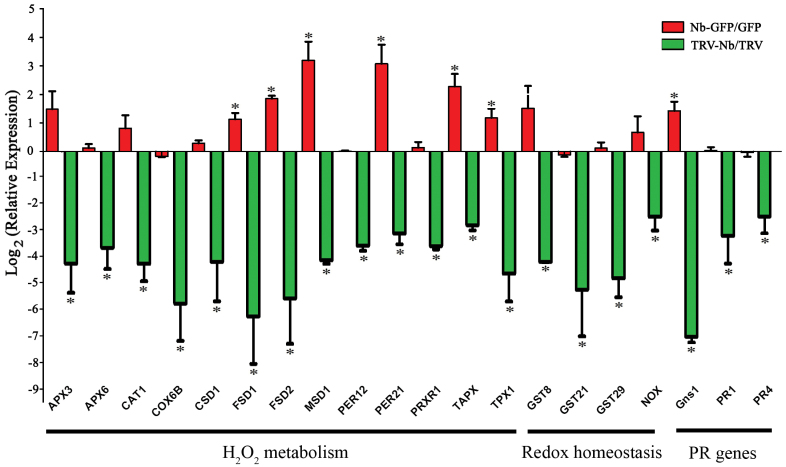
Expression of ROS-responsive genes in *NbNHX1*-overexpressing and silenced plants. Relative expression of each gene was calculated by gene expression in Nb-GFP plants against that in GFP plants (above line), or gene expression in TRV-Nb plants against that in TRV plants (below line). Biparametric output was displayed with log_2_ (relative expression) and the name of each gene. *APX3*, ascorbate peroxidase 3 gene; *APX6*, ascorbate peroxidase 6 gene; *CAT1*, catalase 1 gene; *COX6B*, cytochrome *c* oxidase 6b gene; *CSD1*, copper/zinc superoxide dismutase 1 gene; *FSD1*, Fe superoxide dismutase 1 gene; *FSD2*, Fe superoxide dismutase 2 gene; *MSD1*, manganese superoxide dismutase 1 gene; *PER12*, peroxidase 12 gene; *PER21*, peroxidase 21 gene; *PRXR1*, secretory peroxidase gene; *TAPX*, l-ascorbate peroxidase gene; *TPX1*, thioredoxin-dependent peroxidase 1 gene; *GST8*, glutathione transferase 8 gene; *GST21*, glutathione transferase 21 gene; *GST29*, glutathione transferase 29 gene; *NOX*, NADPH oxidase gene; *Gns1*, beta-1,3-glucanase 1 gene; *PR1*, pathogenesis-related protein 1 gene; *PR4*, pathogenesis-related protein 4 gene. Data are means ±SE (*n*=3 independent *NbNHX1*-silenced or overexpressing lines, respectively). The asterisks on the bars indicate significant differences from the control plants in the same treatment at *P*≤0.05. (This figure is available in colour at *JXB* online.)

The expression of 20 ROS-responsive genes in *NbNHX1*-overexpressing (Nb-GFP-5, Nb-GFP-6, and Nb-GFP-9, Supplementary Fig. S4G at *JXB* online) or silenced (TRV-Nb-10, TRV-Nb-16, and TRV-Nb-25, Supplementary Fig. S2C) *N. benthamiana* was examined. These genes were selected from the unigene database in the Sol genomics network (http://solgenomics.net/), and were divided into three categories. The first category consisted of genes related to H_2_O_2_ metabolism, including ascorbate peroxidase genes, *APX3*, *APX6*, and *TAPX*; a catalase gene, *CAT1*; a cytochrome *c* oxidase gene, *COX6B*; superoxide dismutase genes, *CSD1*, *FSD1*, *FSD2*, and *MSD1*; and peroxidase genes, *PER12*, *PER21*, *PRXR1*, and *TPX1*. Category II were genes related to redox homeostasis, including *GST8*, *GST21*, *GST29*, and *NOX*. Category III were PR (pathogenesis-related) genes, including *PR1*, *PR2* (*Gns1*), and *PR4*. The results showed that overexpression of *NbNHX1* increased expression of *FSD1*, *FSD2*, *MSD1*, *PER21*, *TAPX*, *TPX1*, and *Gns1* in Nb-GFP plants, while silencing of *NbNHX1* decreased the expression of all genes (Fig. 10).

## Discussion

Thus far, little is known about the function of NHX1 in biotic stresses. In the present study, it was found that endogenous *NbNHX1* silencing led to more serious damage after pathogen inoculation in *N. benthamiana* (Fig. 3). Although the expression of *NbNHX1* was found to be induced by *Ppn* infection (Supplementary Fig. S4A at *JXB* online), the enhanced expression could not compensate the reduction by gene silencing. Furthermore, the finding that ectopic expression of *At/SeNHX1* improved resistance to *Ppn* in *N. benthamiana* confirmed the general characteristic of *NHX1* in plant disease resistance (Fig. 4). The overexpression of *NbNHX1* by PEBV was not used in the present study because endogenous genes can be silenced easily by this system (Constantin *et al.*, 2004). It should be noted that although all the *NHX1*-transformed tobacco plants succumbed after *Ppn* attack, the alleviated oxidative damage caused by pathogen infection was important for agriculture production, in that potentially use of the system could buy the time for subsequent chemical prevention.

It has been reported that NHX1 transports both Na^+^ and K^+^ cations with similar affinities (Yamaguchi *et al.*, 2003). Although the mechanism of the regulation of potassium transportation by NHX1 is still unclear (Martinoia *et al.*, 2007), some evidence supports that NHX1 can mediate potassium compartmentation in vacuoles (Apse *et al.*, 2003; Leidi *et al.*, 2010). In the present study, the effect of exogenous Na^+^ or K^+^ application on the activity of NHX1 in transporting protons was investigated. In particular, the measurement simulated the intracellular ionic environment with 100mM potassium gluconate (Chen *et al.*, 2011*b*). As an increasing concentration of NaCl was added to the measuring buffer, TRV plants exhibited enhanced H^+^ efflux in the vacuole; whereas TRV-Nb plants exhibited unchanged H^+^ efflux in vacuoles, which was due to the silencing of *NbNHX1* (Fig. 1D). When extra 25mM or 50mM KCl was added into the measuring buffer, the net H^+^ efflux in the vacuoles remained unchanged in TRV plants (Fig. 1E), which may be attributed to little change in the K^+^ concentration in the measuring buffer. These results suggest that because of a high concentration of K^+^ in the cytoplasm, a change in Na^+^ concentration may more easily affect vacuolar proton transport than K^+^.

Notably, it is reported that tonoplast Na^+^/H^+^ antiporters are involved in cytoplasmic acidification in response to microbial elicitors (Viehweger *et al.*, 2002), which is known to induce oxidative burst. The present results support that the tonoplast-localized Na^+^/H^+^ exchangers regulating vacuolar pH are involved in cellular oxidative events. The concentration of superoxide in the endosomes depends on many factors, including lumen pH (Lamb *et al.*, 2009). Due to the proton dependence of dismutation, decomposition of superoxide is prolonged ~10-fold for every 10-fold decrease in the proton concentration between pH 6 and 14 (Valentine and Curtis, 1975). It has also been confirmed that the contents of superoxide in vesicles improve along with increasing pH, from 4.4 μM superoxide in pH 5.0 to 30 μM in pH 8.0 (Mumbengegwi *et al.*, 2008). In addition, superoxide produced in endosomes always affects the cellular oxidative state, since superoxide can diffuse easily via transmembranes of endosomes (Brunetti *et al.*, 2011), and the tonoplast and other cellular membranes are quite permeable to Н_2_О_2_ (Andreev, 2012). Therefore, it is understandable that increased pH in the vacuole due to overexpression of *NbNHX1* resulted in an improved cellular oxidation level in tobacco (Figs 6A, 7), and silencing of *NbNHX1* reducing the pH in the vacuole led to a decreased cellular oxidation state (Figs 6B, 8). The conclusion that the pH in the vacuole affected the cellular oxidation state is consistent with the results of Mumbengegwi *et al.* (2008) and Pradedova *et al.* (2011), which implies that either accumulation of superoxide in the vacuole, or its being shielded from dismutation, is based on proton transportation across the tonoplast.

The change in cellular oxidation state triggers physicochemical responses, which results in a rapid re-establishment of redox homeostasis (Luthje *et al.*, 2009). In the present study, an enhanced cellular oxidation level was also found to lead to a larger NAD(P) (H) pool and higher expression of redox homeostasis-related genes in *NbNHX1*-overexpressing plants (Figs 7, 9, and 10), and vice versa in *NbNHX1*-silenced plants (Figs 8, 9, 10). The NAD(P) (H) components are important molecules in plant response to oxidative stress (Foyer and Noctor, 2003), and the content of NADPH in particular can be significantly increased when cells suffer oxidative damage (Valderrama *et al.*, 2006; Y.P. Wang *et al.*, 2014). It has also been reported that increasing the content of one of the NAD(P) (H) components results in greater contents of both the oxidized and reduced forms of NADs as a larger NAD(P) (H) pool (Hayashi *et al.*, 2005). The evidence also confirms that the NAD(P) (H) pool can be regulated by the cellular oxidation level (Cueno *et al.*, 2014). The present study also found that transformation of *NbNHX1* conferred a higher cellular oxidation level on tobacco, resulting in a larger NAD(P) (H) pool (Figs 7, 9). In addition, increased vacuolar H^+^ efflux might boost proton supplementation for NADPH oxidation mediated by the tonoplast-localized NOX, which would speed up NADP(H) recycling. Therefore, it is understandable that the *NbNHX1*-overexpressing plants with a higher cellular oxidation level exhibited a larger NAD(P) (H) pool. It should be noted that the NAD(P) (H) components in GFP plants were measured after 3 d agroinfiltration, and those in TRV plants were detected after 4 weeks agroinfiltration for gene silencing, and hence there were some differences in NAD(P) (H) levels between GFP and TRV plants (Fig. 9).

Ascorbate peroxidases (APXs), catalases (CATs), and peroxidases (PODs) are very important enzymes for H_2_O_2_ detoxification, cytochrome *c* oxidases (COXs) are important for reduction of O_2_ to H_2_O, and superoxide dismutases (SODs) catalyse the dismutation of O_2-_ into H_2_O_2_, which are all associated with cellular ROS homeostasis (Garg and Manchanda, 2009). In this study, it was found that the genes involved in H_2_O_2_ metabolism: *APX* genes (*APX3*, *APX6*, and *TAPX*), *CAT1*, *COX6B*, *POX* genes (*PER12*, *PER21*, and *TPX1*), and *SOD* genes (*CSD1*, *FSD1*, *FSD2*, and *MSD1*) were regulated by the cellular oxidation level in Nb-GFP or TRV-Nb plants (Fig. 10). A changed cellular oxidation state served as a signal to trigger the expression of antioxidant-related genes (Liu *et al.*, 2010). For example, it is reported that sulphur dioxide (SO_2_) can improve cellular ROS levels, leading to higher expression of genes encoding SODs (*CSD1*, *CSD2*) and PODs (*POD*) (Li and Yi, 2012). However, the gene expression of the ROS scavenging network in *Arabidopsis* indicates that there are more complicated molecular events in plant response to oxidative damage (Mittler *et al.*, 2004). A mutant (knockout of *SOD* gene) affecting production of H_2_O_2_ exhibits down-regulated expression of nearly all ROS-responsive genes in *Arabidopsis* (Mittler *et al.*, 2004), which to some extent supports that expression of ROS-responsive genes decreased in *NbNHX1*-silenced plants with a lower cellular oxidation level (Figs 8, 10). Another possible reason is that *NbNHX1* silencing led to a significant decrease in cellular pH which was associated with classic apoptotis of acidified cells and DNA cleavage (Boyle *et al.*, 1997), and hence TRV-Nb plants exhibited decreased expression of most genes. In addition, PR genes (*PR1*, *Gns1*, and *PR4*) were regulated by the cellular oxidation level in *NbNHX1*-silenced or overexpressing plants (Fig. 10). It has also been reported that in *ZmSIMK1* transgenic tobacco regulating cellular ROS promotes transcription of PR genes such as *PR1*, *PR2*, and *PR4* (L. Wang *et al.*, 2014).

H_2_O_2_ plays different roles in plant defence against biotrophic and necrotrophic pathogens. Plants rely on oxidative burst against biotrophic pathogens but are dependent on the alleviation of H_2_O_2_ against necrotrophic pathogens (Glazebrook, 2005). It was further investigated whether *NHX1* transgenic tobacco plants with enhanced abilities to alleviate H_2_O_2_ were more sensitive to biotrophic pathogens. As shown in Supplementary Fig. S6 at *JXB* online, the leaves of Se-YFP and At-YFP plants, along with those of YFP plants, were infected by the biotrophic pathogen *Pseudomonas syringae* pv. *maculicola* ES4326 (Moeder *et al.*, 2005). The bacterial colonies were measured at 0, 72, and 96 hpi, and the results showed that *SeNHX1* and *AtNHX1* transgenic plants and control tobacco plants exhibited comparable growth of bacteria (Supplementary Fig. S6). It is assumed that the enhanced antioxidative system in *NHX1* transgenic tobacco impaired the diffusion of ROS produced by *Ppn* infection but could not inhibit the oxidative burst induced by biotrophic pathogens. Although tests in more plant species are still needed to investigate further NHX1 function in disease resistance, the present results imply that NHX1 has the potential to be used to increase both salt tolerance and disease resistance in plant.

## Supplementary data

Supplementary data are available at *JXB* online.

Figure S1. Screening the distinctive sequence of *NbNHX1* for gene silencing.

Figure S2. Virus-induced gene silencing and ectopic expression in *N. benthamiana*.

Figure S3. *In situ* calibration curve and pH quantification test.

Figure S4. The relative expression of *NHX1* in different tobacco genotypes.

Figure S5. Vacuolar H^+^ net fluxes in *NbNHX1*-silenced *N. benthamiana* under 0 and 1.5mM ATP or PPi treatment.

Figure S6. Responses of *NHX1* transgenic tobacco to ES4326.

Table S1. The primers of ROS-responsive genes used in real-time PCR.

Supplementary Data

## Abbreviations:

At/Se-YFP plant: pCAPE2-At/SeNHX1-YFP vector-transformed tobacco
DR: CellROX deep red reagent
Fdox/Fdred: oxidized/reduced ferredoxin
FSC: forward and side scatter value
GFP: green fluorescent protein
GFP plant: 35S::GFP cassette-transformed tobacco
GSSG/GSH: oxidized/reduced glutathione
hpi: hours post-inoculation
Nb-GFP: 35S::NbNHX1-GFP cassette-transformed tobacco
NHX1: Na^+^/H^+^ exchanger 1
NMT: non-invasive micro-test electrophysiological technology
NOX: NADPH oxidase
PEBV: Pea early browning virus
Ppn: *Phytophthora parasitica* var. *nicotianae*
PR: pathogenesis related
RACE: rapid amplification of cDNA ends
ROS: reactive oxygen species
TRV: *Tobacco rattle virus*
TRV plant: pTRV2 empty vector-transformed tobacco
TRV-Nb plant: pTRV2-NbNHX1 vector-transformed tobacco
YFP: yellow fluorescent protein
YFP plant: pCAPE2 empty vector-transformed tobacco.
