# Supplementary material for: Na+/H+ exchanger 1 participates in tobacco disease defence against Phytophthora parasitica var. nicotianae by affecting vacuolar pH and priming the antioxidative system
Source: J Exp Bot. 2014 Aug 28;65(20):6107–22. doi: 10.1093/jxb/eru351 (PMC4203143; doi:10.1093/jxb/eru351)
Supplement: Supplementary Data [file supp_65_20_6107__index.html]

Na+/H+ exchanger 1 participates in tobacco disease defence against Phytophthora parasitica var. nicotianae by affecting vacuolar pH and priming the antioxidative system — Na+/H+ exchanger 1 participates in tobacco disease defence against Phytophthora parasitica var. nicotianae by affecting vacuolar pH and priming the antioxidative system — Supplementary Data 

# Na+/H+ exchanger 1 participates in tobacco disease defence against *Phytophthora parasitica* var. *nicotianae* by affecting vacuolar pH and priming the antioxidative system

## Supplementary Data

Data files

**Files in this Data Supplement:**

- Supplementary Data - Supplementary Data
