# Supplementary material for: Na+/H+ exchanger 1 participates in tobacco disease defence against Phytophthora parasitica var. nicotianae by affecting vacuolar pH and priming the antioxidative system
Source: J Exp Bot. 2014 Aug 28;65(20):6107–22. doi: 10.1093/jxb/eru351 (PMC4203143; doi:10.1093/jxb/eru351)
Supplement: Supplementary Data [file supp_eru351_jexbot131185_file001.pdf]

Title: NHX1 participates in tobacco disease defense against *Phytophthora parasitica* var. *nicotianae* by affecting vacuolar pH and priming the anti-oxidative system

Xianyang Chen, Hexigeduleng Bao, Jie Guo, Weitao Jia, Fang Tai, Lingling Nie, Ping Jiang, Juanjuan Feng, Sulian Lv, Yinxin Li *

Supplementary Figure S1. Screening distinctive sequence of *NbNHX1* for gene silencing. A, EST information of *NbNHX3* cloned from *N. benthamiana*. The yellow mark indicates the same sequence between unigene of SGN-U521516 and the EST of *NbNHX3*; red mark presents the same sequence between unigene of SGN-U518331 and the EST of *NbNHX3*. B-C, The sequence alignment among *NbNHX1* (Nb1), unigene *NbNHX2* (Nb2, ID: SGN-U515339), and unigene *NbNHX3* (Nb3, ID: SGN-U518331). The underline sequence indicates the distinctive 263 bp sequence from *NbNHX1* used for gene silencing.

Supplementary Figure S2. Virus inducing gene silencing and ectopic expression in *N. benthamiana*. A, TRV inducing gene silencing in *N. benthamiana*. Diagrams illustrate the pTRV2 derivative clone vectors used in this study. B, Phenotypes of *N. benthamiana* at 28 days post inoculation with TRV carrying a conserved fragment of a phytoene desaturase gene. C, Expression of *NbNHX1* in *NbNHX1* silenced tobaccos (N1-N30). D, Wilt spot area in *NbNHX1* silenced tobaccos (N1-N30). E, PEBV mediating ectopic gene expression in *N. benthamiana*. Diagrams illustrate the pCAPE2 derivative clone vectors used in this study. F, Phenotypes of *N. benthamiana* at 28 days post inoculation with PEBV carrying a conserved fragment of a phytoene desaturase gene. G, Expression of *NHX1* in *SeNHX1* transgenic tobaccos (S1-S30) and *AtNHX1* transgenic tobaccos (A1-A30). H, Wilt spot area in *SeNHX1* transgenic tobaccos (S1-S30) and *AtNHX1* transgenic tobaccos (A1-A30).

Supplementary Figure S3. *In situ* calibration curve and pH quantification test. A, The calibration curve was performed by plotting the ratio of emission fluorescence (505-550 nm) excited with 458 or 488 nm, against the pH of equilibration buffers. B,

Vacuolar pH quantification under TEST1 (pH 5.1) and TEST (pH 5.4) treatment. Data are means ± SE (*n* = 60 cells from 6 leaves).

Supplementary Figure S4. The relative expression of *NHX1* in different genotype tobacco. A, The relative expression of *NbNHX1* at 0, 24, 36 and 48 hpi. B, The relationship between relative expression of *NbNHX1* and vacuole H^+ net flux. C, The relative expression of *NbNHX1* in NS1-NS6. The relative expression of *NHX1* in S31-S36 (D), A31-A36 (E) and NOE1-NOE6 (F). G, The relative expression of *NbNHX1* in 6 Nb-GFP plants. The relative expression was calculated by actual expression of *NHX1* against that in untreated, or TRV plants or GFP plants.

Supplementary Figure S5. Vacuolar H^+ net fluxes in *NbNHX1* silenced *N. benthamiana* under 0 and 1.5 mM ATP or *PPi* treatment. Dynamic curves of transient H^+ net fluxes: normal condition plus supply of ATP (A) or *PPi* (B). C-D, Mean of vacuolar H^+ net fluxes corresponding to (A-B) during the measuring periods. E, The changes of vacuolar H^+ flux (ΔH^+ flux) after ATP or *PPi* supply in TRV or TRV-Nb plants. The value obtained from NMT indicates net ion flux; the positive values of H^+ flux in the figures represent cation efflux or anion influx, and *vice versa*. TRV represents pTRV2 empty vector transformed tobaccos (control plants). TRV-Nb represents pTRV2-NbNHX1 vector transformed tobaccos (*NbNHX1* silenced plants). Data are means ± SE (*n* = 6 vacuole per *NbNHX1* silenced line, 6 independent *NbNHX1* silenced lines). The asterisks on the bars indicate significant differences from the TRV plants in the same treatment at $P \leq 0.05$.

Supplementary Figure S6. Responses of *NHX1* transgenic tobaccos to ES4326. Infection experiments were performed with 6-week-old plants of Se/At-YFP transgenic and YFP plants mediated by PEBV. 30 leaves were collected to examine the growth of the pathogen at 0, 72, and 96 hpi. YFP means pCAPE2-YFP vector transformed tobaccos. Se/At-YFP represents pCAPE2-At/SeNHX1-YFP vector transformed tobaccos. Data are means ± SE (*n* = 30 leaves from 30 independent transgenic lines). The asterisks on the bars indicate significant differences from the YFP plants in the same treatment at $P \leq 0.05$.

Supplementary Table S1. The primers of ROS responsive genes used in real-time PCR.

Supplementary Figure S1

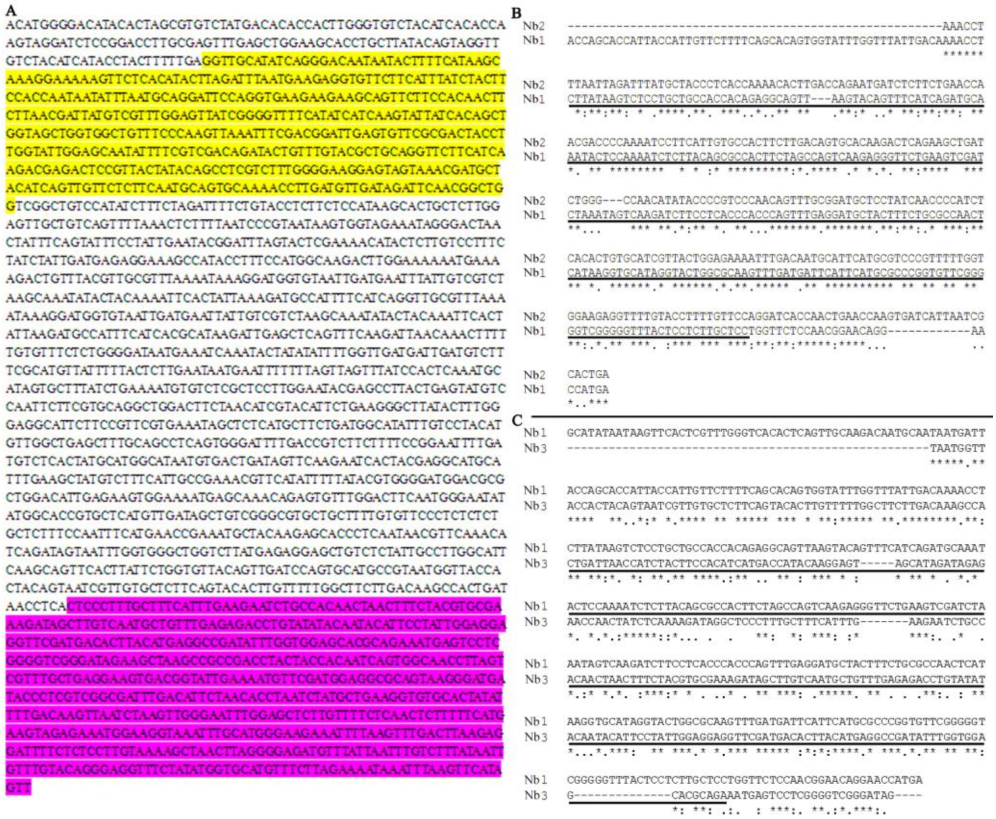

Supplementary Figure S2

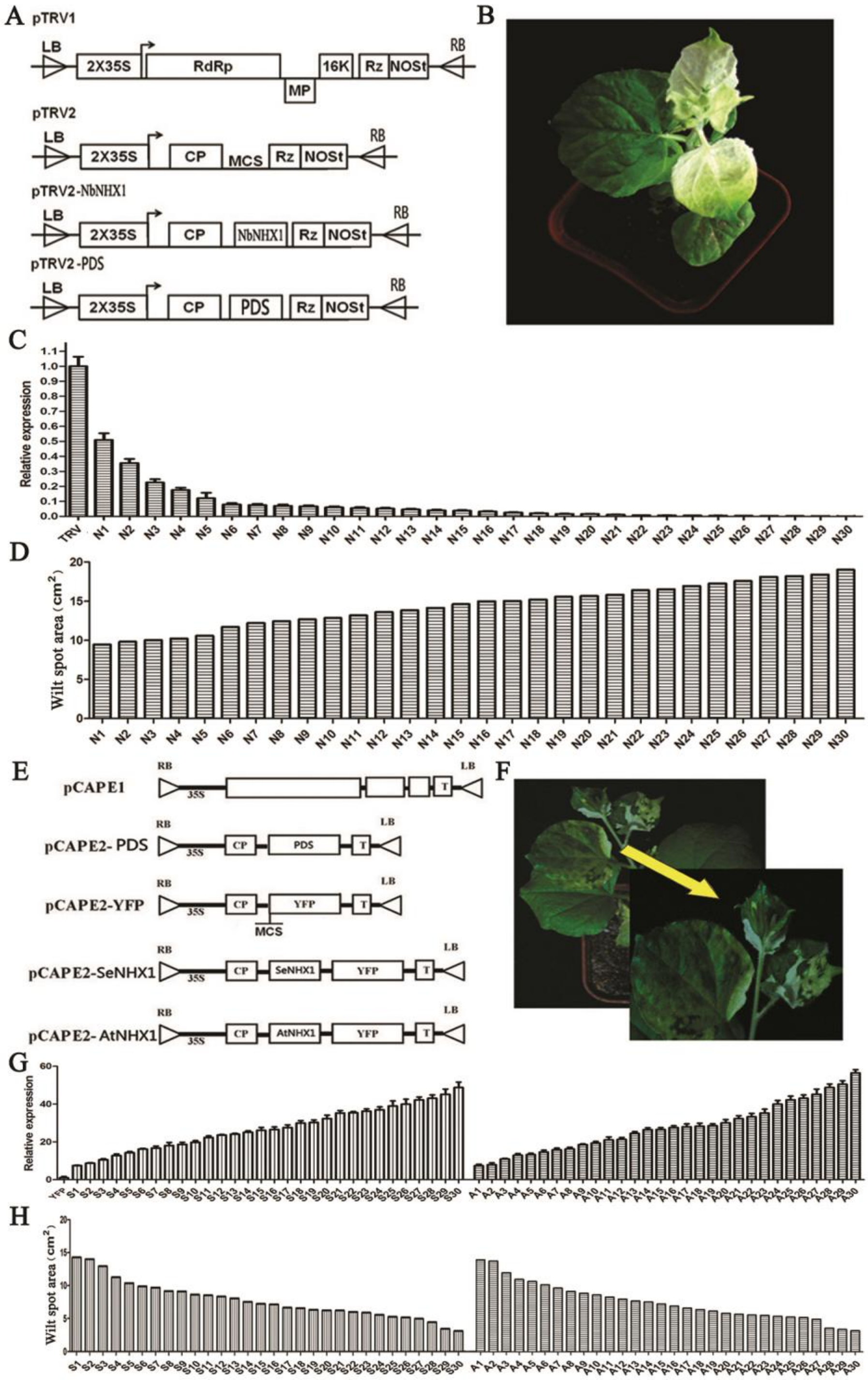

Supplementary Figure S3

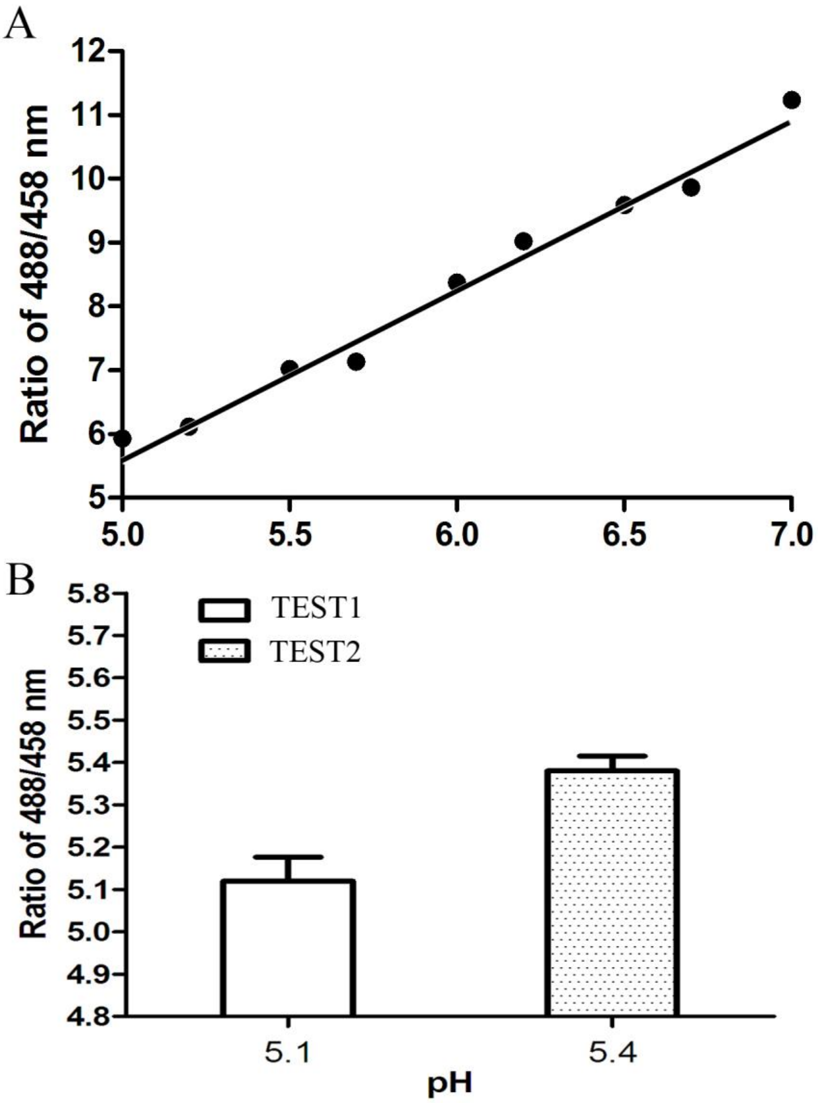

Supplementary Figure S4

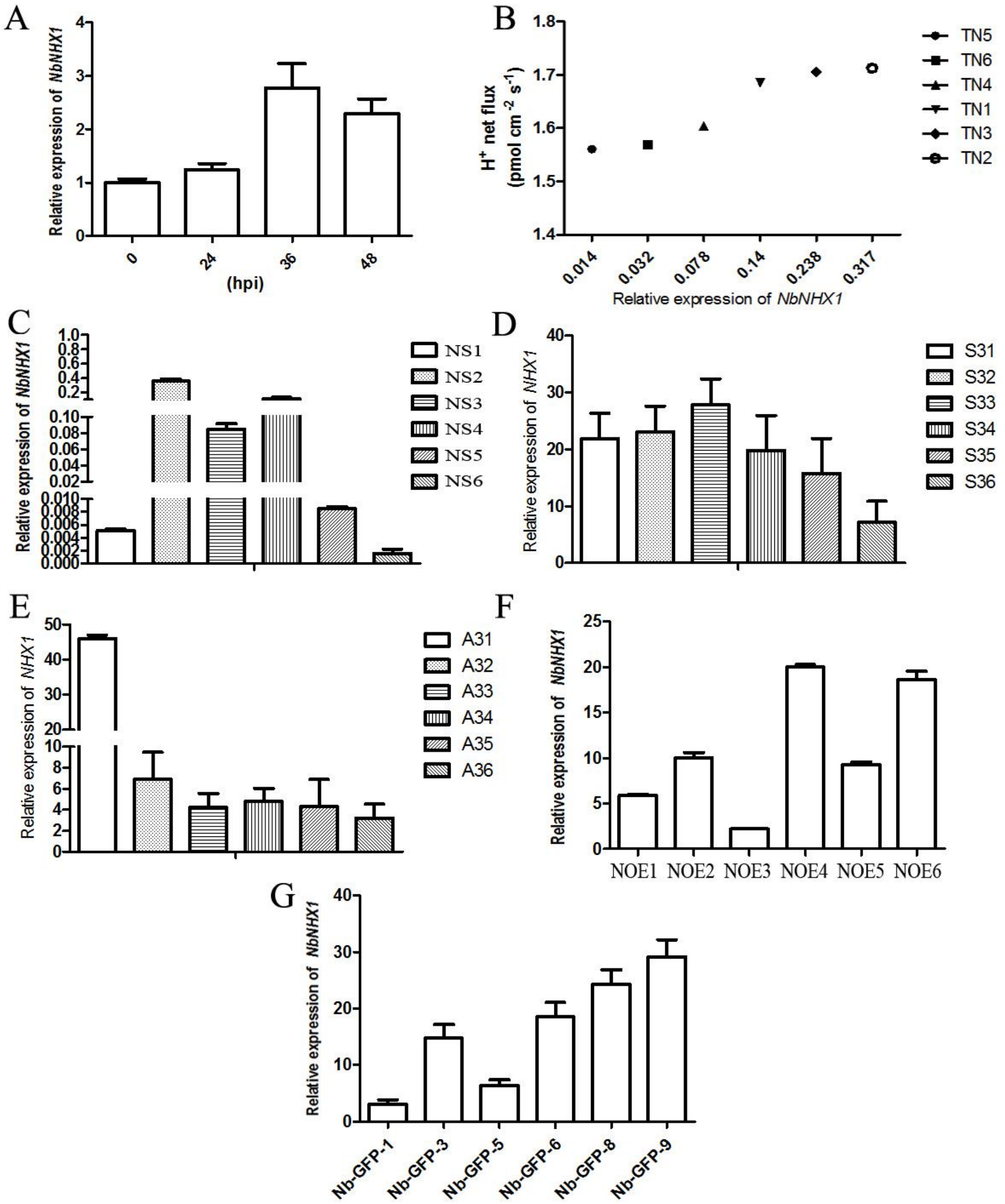

Supplementary Figure S5

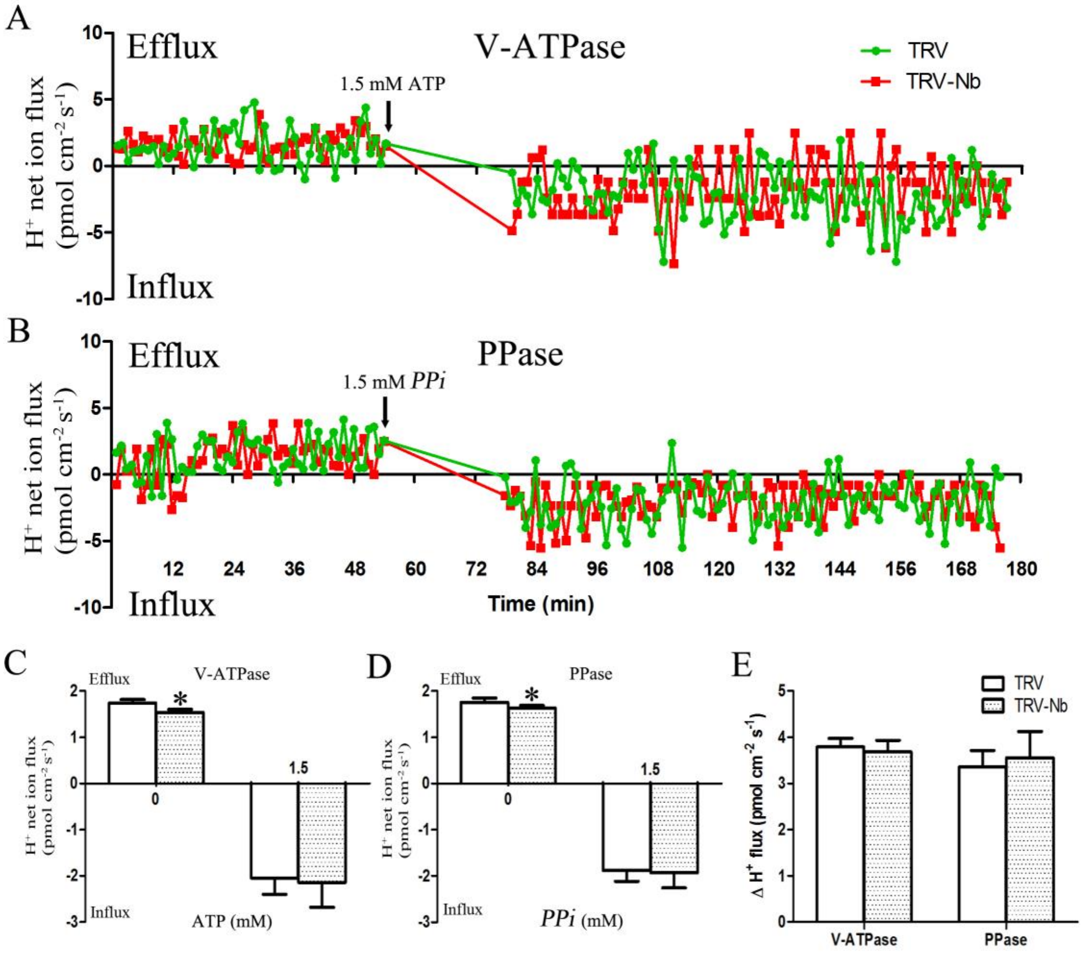

Supplementary Figure S6

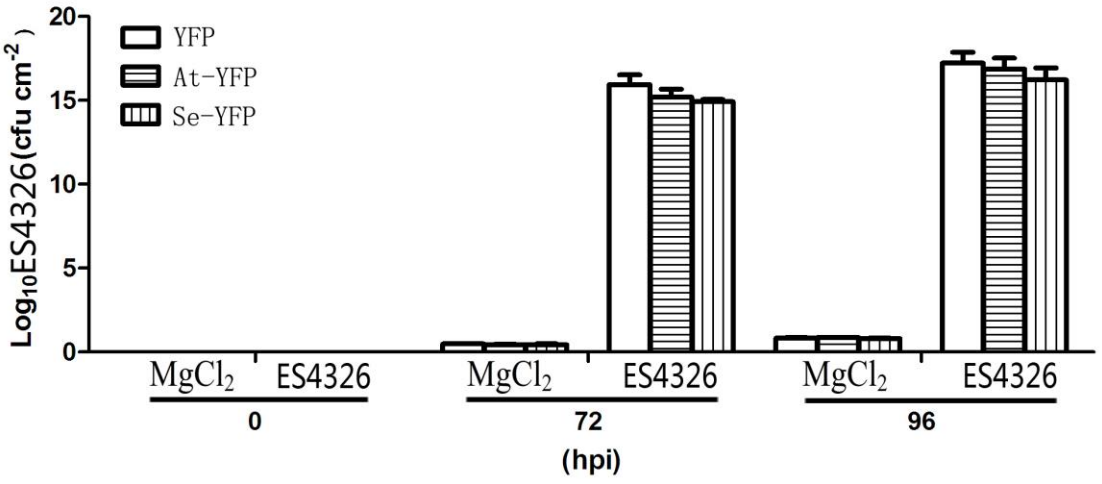

Supplementary Table S1.

Gene name	Unigene No.	Primers
TPX1	SGN-U505985	F 5' GCCGTTGGTGACATCATTCCC 3'
		R 5' CCTTTAGATTTCAACTCGTCAGC 3'
COX6B	SGN-U511126	F 5' CAGGTTCCCAACAACCAATCAAA 3'
		R 5' CCCATTCTCCCTCTGCTCGTT 3'
CSD1	SGN-U514519	F 5' GCAGCAGTGAAGGTGTTAGTGGCA 3'
		R 5' GGATTGTGGACCAGCAAGAGGAAT 3'
CAT1	SGN-U518840	F 5' CATACAAGTACCGTCCGTCAAGTGC 3'
		R 5' GAAAAACCCTTTGGCACTAGCACCTC 3'
TAPX	SGN-U506407	F 5' TCCCTCAAATGCCTCCAATCCT 3'
		R 5' GCATTTCGGCGAGGCTAACG 3'
PER21	SGN-U506123	F 5' TTGACAATGAAGCCCAGGTGA 3'
		R 5' CTCCTCCGCAGATTGATTGG 3'
PER12	SGN-U515270	F 5' CTGCTAGGGACTCTGTTGTCTTGACT 3'
		R 5' CGACGGAGGACGACTGTGGTATT 3'
NOS	SGN-U518155	F 5' TAAGACAACACGCCTTGGATT 3'
		R 5' GCCTGCCTTACTATTTGCCTC 3'
APX6	SGN-U521022	F 5' GGGTTTGGGAATCCAGTTGTC 3'
		R 5' TGTCAACAAGTTTGGTATAGGCATT 3'
PRXR1	SGN-U509102	F 5' TTTGCGGAGGACAATTCTGGC 3'
		R 5' CCTGGACACTCCCTTTCAACAG 3'

APX3	SGN-U515717	F 5' GGAAGGACGGTTGCCCGATGCT 3'
		R 5' TGTCTGTGGGAAGTTTCAGTAGGC 3'
FSD2	SGN-U508368	F 5' CTGGTTCTGGCACAATAACGGCT 3'
		R 5' TCCTCCTCCGTTGGGCTTCA 3'
FSD1	SGN-U508369	F 5' TGGCTCGCATACAAACCTGAA 3'
		R 5' CCACGACACGAGCTTCTCCATAA 3'
MSD1	SGN-U508400	F 5' GTGAGCAGACGGACCTTAGCAACA 3'
		R 5' CGTGACCTCCGCCGTTGAATT 3'
GST8	SGN-U513459	F 5' GCAGTATGTATGGGATGAGGGC 3'
		R 5' TTCACTGCATATTCATGTACGGTATT 3'
GST21	SGN-U513460	F 5' GGTTCTGGGCTGATTACACTGAC 3’
		R 5' CCAAAGTAAGGTTTGTCTCCCAAT 3’
GST29	SGN-U511196	F 5' CAGCCAAGGGCAATGTCCAAGTA 3'
		R 5' GTTCTGGTCCATCTATTCTCCCTTCT 3'
PR1	SGN-U512904	F 5' TCAACTCAACGCCGCTGGTG 3'
		R 5' GAATCAAAGGGCTGTTGCTCTTCA 3'
PR4	SGN-U508784	F 5' TTTCTGTGATGCTGCTGGACC 3'
		R 5' GCCTTGCTGATAGCCCACTCC 3'
Gns1	SGN-U515120	F 5' GAAAGCCTGGACCTATTGAAACC 3'
		R 5' GCAGTAGCATTAGTTTCAACCGAAC 3'
